# Ten-year experience with standardized non-operating room anesthesia with Sevoflurane for MRI in children affected by neuropsychiatric disorders

**DOI:** 10.1186/s12871-019-0897-1

**Published:** 2019-12-18

**Authors:** Silvia Mongodi, Gaia Ottonello, Raffaelealdo Viggiano, Paola Borrelli, Simona Orcesi, Anna Pichiecchio, Umberto Balottin, Francesco Mojoli, Giorgio Antonio Iotti

**Affiliations:** 1 Anesthesia and Intensive Care, Rianimazione 1 Fondazione IRCCS Policlinico S. Matteo, 27100 Pavia, Italy; 2 Anaesthesia and Intensive Care, Merate, DEA ASST Lecco, Lecco, Italy; 3 Department of medical-surgical, diagnostic and paediatric sciences, University of Pavia, Pavia, Italy; 4 Department of Public Health, Experimental and Forensic Medicine, Unit of Biostatistics and Clinical Epidemiology, University of Pavia, Pavia, Italy; 5 Child Neurology and Psychiatry Unit, IRCCS Mondino Foundation, Pavia, Italy; 6 Child Neurology and Psychiatry Unit, Department of Brain and Behavioural Sciences, University of Pavia, Pavia, Italy; 7 Neuroradiology department, IRCCS Mondino Foundation, Pavia, Italy; 8 Department of Brain and Behavioural Sciences, University of Pavia, Pavia, Italy

**Keywords:** Non-operating room anesthesia, MRI sedation, Neuropsychiatric disorders anesthesia, Sevoflurane, NORA

## Abstract

**Background:**

Children require anesthesia for MRI to maintain immobility and reduce discomfort; clear indications about the best anesthesiologic management are lacking and each center developed its own protocol. Moreover, children with neuropsychiatric disorders more likely require sedation and are described in literature as more prone to general and respiratory complications. Aim of this study was to analyze the applicability of a sevoflurane-based approach, to describe general and respiratory complications and to identify risk factors in a pediatric neuropsychiatric population.

**Methods:**

Retrospective cohort study, university Hospital (January 2007–December 2016). All the 1469 anesthesiologic records of children addressed from Neuropsychiatric Unit to undergo MRI under general anesthesia were analyzed; 12 patients equal or older than 18-year-old were excluded. We identified post-hoc nine macro-categories: static encephalopathies, metabolic/evolutive encephalopathies, epileptic encephalopathies, neuromuscular diseases, autistic spectrum disorders, migraine, psychiatric disorders, intellectual disabilities, others. A logistic regression model for events with low frequency (Firth’s penalized likelihood approach) was carried out to identify the mutually adjusted effect among endpoints (complications) and the independent variables chosen on the basis of statistical significance (univariate analysis, *p* ≤ 0.05) and clinical judgment.

**Results:**

Of 1457 anesthesiologic records (age 4.0 (IQR 2.0 to 7.0) year-old, males 891 (61.2%), weight 17.0 (IQR 12.0 to 24.9) kg), 18 were cancelled for high anesthesiologic risk, 50 were cooperative, 1389 were anesthetized. A sevoflurane-based anesthesia was feasible in 92.3%; these patients required significantly less mechanical ventilation (8.6 vs. 16.2%; *p* = 0.012). Complications’ rate was low (6.2%; 3.1% respiratory). The risk for general complications increases with ASA score > 1 (OR 2.22, 95 CI% 1.30 to 3.77, *p* = 0.003), male sex (OR 1.73, 95% CI 1.07 to 2.81, *p* = 0.025), multi-drug anesthesia (OR 2.98, 95 CI% 1.26 to 7.06, *p* = 0.013). For respiratory complications, it increases with ASA score > 1 (OR 2.34, 95 CI% 1.19 to 4.73, *p* = 0.017), autumn-winter (OR 2.01, 95 CI% 1.06 to 3.78, *p* = 0.030), neuromuscular disorders (OR 3.18, 95 CI% 1.20 to 8.41, *p* = 0.020). We had no major complications compromising patients’ outcome or requiring admission to ICU.

**Conclusions:**

Sevoflurane anesthesia is feasible and safe for children affected by neuropsychiatric disorders undergoing MRI. Specific risk factors for general and respiratory complications should be considered.

## Background

Pediatric patients frequently require sedation for medical procedures to reduce pain and stress, or to maintain immobility. In the case of magnetic resonance imaging (MRI), image quality is significantly improved by complete stillness [1].

Since 1985, scientific societies emanated guidelines to perform pediatric non-operating room anesthesia (NORA), in order to reduce complications [2]. In the last International Guidelines update in 2016 [3] and in the 2019 clinical practice statement from the European Society of Paediatric Anaeshesiology [3], many aspects of pediatric NORA are detailed, as anesthesia record, monitoring, setting and dedicated personnel’s competences in function of the expected level of sedation. However, no clear indication on the most appropriate pharmacological approach is reported [3–5] and each center developed its own protocol [6–17]. Different pharmacological approaches have specific drawbacks, as high failure rate and poor predictability (chloral hydrate [6–9], dexmedetomidine [10, 11], thiopental [9], midazolam [7]), hemodynamic side effects (dexmedetomidine [10, 11]), prolonged awakening (midazolam [7], chloral hydrate [6–9]) and respiratory drive depression (propofol [11–15]). Sevoflurane presents extremely good results in term of success of sedation, safety and manageability, but is mainly described in infants [16, 17].

Moreover, children affected by neuropsychiatric disorders are considered at higher risk of anesthesiologic complications [18]. A higher rate of pulmonary complications was in fact described by Cravero et al. [13] in children with a higher American Society of Anesthesiology (ASA) status; a large proportion of this population was affected by neurological disorders. Children affected by developmental disabilities and neurologic disorders seem to be more prone to anesthesia-related respiratory complications when compared to healthy children, notably in terms of airway compromise [19]. This may in part be explained by a reduced oro-pharyngeal airway diameter when compared to general pediatric population [20]. A variety of genetic diseases has been described at higher risk of sedation/anesthesia complications [21]. A higher sensitivity to both inhaled anesthetics [22] and opioids [23] has also been described in cerebral palsy.

Among neuropsychiatric disorders, neuromuscular diseases are considered at the highest risk of perioperative respiratory complications since they may affect respiratory muscles strength either directly by muscle fibres weakening or indirectly by degenerative nerve supply and neuromuscular junction weakness [24–26]. A careful and multidisciplinary pre-anesthesia assessment is in general recommended [27, 28]. Moreover, such diseases are considered at high risk of specific anesthesiologic complications as malignant hyperthermia or rhabdomyolysis, in particular after inhaled anesthetics’ administration [24, 29].

As in other institutions, in our center we systematically applied a standardized approach with sevoflurane-based NORA as described in infants and neonates [16, 17], eventually associated to premedication with midazolam, in spontaneous breathing. However, in literature evidence is lacking on the applicability, rate of complications and risk factors for such anesthesiologic approach in children.

Aim of this study was to analyze a ten-year experience of pediatric NORA for MRI performed with a standardized sevoflurane-based approach in children affected by neuropsychiatric disorders, focusing on applicability of the approach, anesthesia management, rate of general and respiratory complications and risk factors’ identification.

## Methods

Ethical approval for this study (Ethical Committee N° P-20170030606) was provided by the Ethical Committee NAC of Fondazione IRCCS Policlinico S. Matteo University Hospitals, Pavia, Italy (Chairperson Prof M. Cazzola) on December 5th, 2017; the need for written consent was waived by the ethics committee.

We retrospectively enrolled all patients addressed from Pediatric Neuropsychiatric Unit of Fondazione Mondino IRCCS, a university Italian neurological hospital, to undergo MRI under general anesthesia (GA) from January 2007 to December 2016. We excluded from final analysis patients equal or older than 18-year-old. Data were collected from anesthesia records. A simplified classification of neuropsychiatric disorders was performed post-hoc; nine macro-categories were identified: static encephalopathies (cerebral palsy, syndromic-genetic disorders, malformative diseases), metabolic/evolutive encephalopathies (leukodystrophies, grey substance diseases, mitochondrial diseases), epileptic encephalopathies, neuromuscular diseases, autistic spectrum disorders, migraine, psychiatric disorders, intellectual disabilities, others. In particular, we focused our attention on children affected by neuromuscular diseases, being more exposed to respiratory complication [24–30].

### Standardized sevoflurane-based NORA

Anesthesia team is composed by two intensivist-anesthesiologists and one MRI nurse; the hospital is neurological only and no other medical-emergency team is available. Standard practice consists in sevoflurane-based anesthesia induction and maintenance, obtained with sevoflurane inhaled by a reservoir bag mask at 8.0 and 2.5% respectively. Anesthetic gas was delivered by a Drager Fabius© machine and dispersion was prevented by a canopy and aspiration system. If a premedication is indicated, midazolam (0.1 mg/kg administered intramuscularly (IM) 30 min before anesthesia) is administered. Anesthesiologist is free to leave the standard approach, if required according to pre-anesthesia evaluation. After induction, airways’ patency is judged on the basis of respiratory thoracic excursion, peripheral oxygen saturation and expiratory CO_2_ monitoring (capnography) as measured by nasal and/or oral sampling lines. If airways’ patency is judged satisfactory, the patient is maintained in spontaneous breathing, either spontaneously or with an oral cannula, with inhaled sevoflurane by bag mask. Otherwise a laryngeal mask airway (LMA) is electively placed and positive pressure ventilation in a closed circle system started by the same Drager Fabius© machine. Oro-tracheal intubation set is also available. We consider loss of airways patency as a complication in patients with altered capnography or desaturation after the induction phase. A cervical collar is frequently placed to prevent head involuntary movements, eventually impairing image quality. A peripheral vein catheter is placed under GA and glucose 5% 20 ml/h administered to prevent hunger symptoms from fasting at awakening. Italian recommendations for fastening were followed [5]. Vital parameters are continuously monitored by an MRI-compatible monitor and manually noted on anesthesia record every 10 min; all adverse events and associated procedures (i.e. procedures performed under GA after MRI) are also noted. Discharge criteria are a child fully awake or equal to pre-induction status and stable vital parameters.

### Statistical analysis

Mean values and standard deviations (SD) or median and interquartile range (IQR) were used for the quantitative variables, and percentages for the categorical ones. Normal distribution was assessed by Shapiro-Wilk test.

Primary end-point was the applicability of the sevoflurane-based approach, as measured by the percentage of anesthesia conducted with the standardized approach.

Secondary end-points were: to identify differences between standard and non-standard anesthesia, in terms of patients’ characteristics, anesthesia characteristics and need of mechanical ventilation; to describe anesthesiologic management in this specific population in terms of anesthetic drugs for induction and maintenance, need of mechanical ventilation and associated factors, indication to additional procedures during MRI anesthesia, length of anesthesia and time for full recovery; to measure the rate of general and respiratory complications and associated risk factors.

Comparisons among the categorical variables were evaluated with the Pearson chi-square test; Fisher’s exact test and Student’s T Test for independent data or Wilcoxon/Mann-Whitney U-test were used for quantitative variables.

To evaluate the associations between endpoint variable (development of complications during the procedure) and the patients characteristics / anesthesia management, odds ratios (ORs) and the corresponding 95% confidence interval (CI) were calculated. Given the limited number of cases with complications (general and respiratory complications), a logistic regression model for events with low frequency (Firth’s penalized likelihood approach) was carried out to identify the mutually adjusted effect among endpoints and the independent variables chosen on the basis of the statistical significance (univariate analysis, *p* ≤ 0.05) and of the clinical judgment; age and sex were used as adjusting variables. *P*-value ≤0.05 was considered significant (two-sided). All the analyses were conducted with STATA/SE for Macintosh, version 14.2.

## Results

### Population

**Fig. 1:**
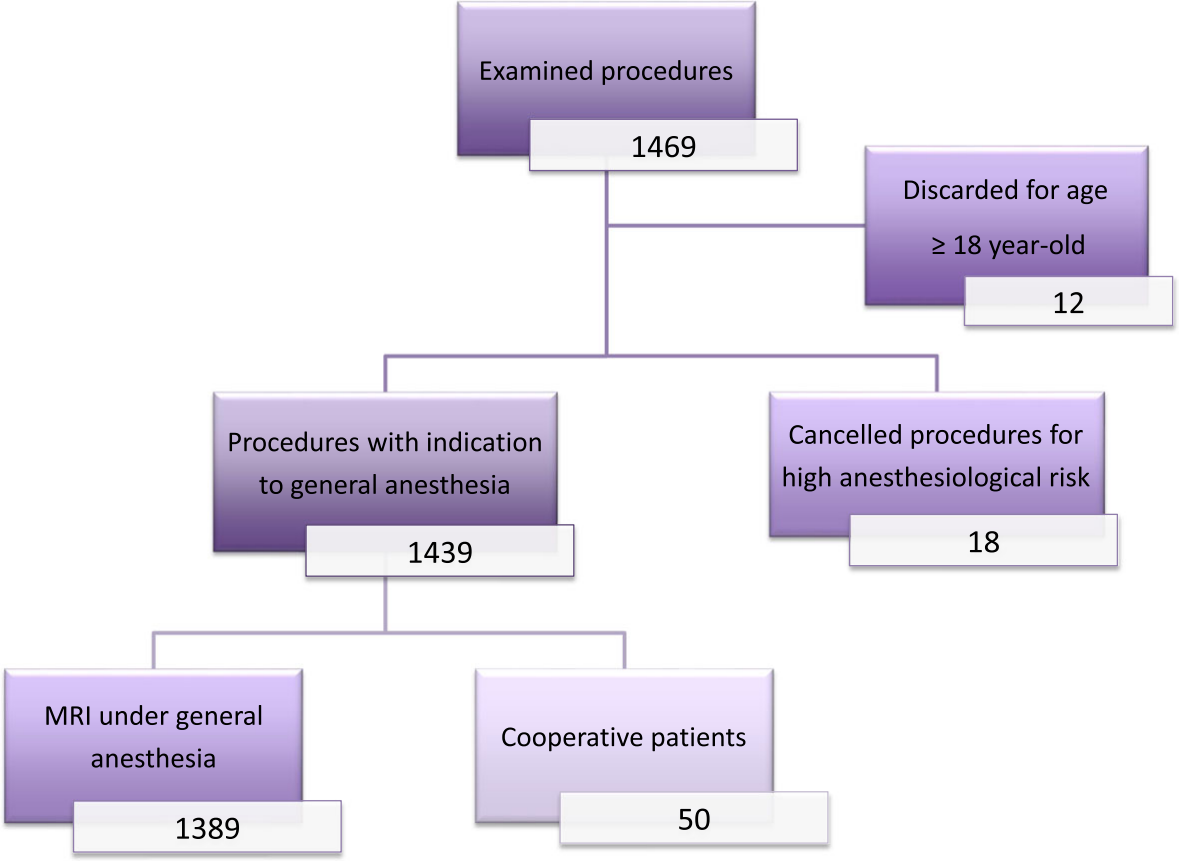
Diagram of patient recruitment

We analyzed 1457 records of 1268 patients addressed by Pediatric Neuropsychiatric Unit, having excluded 12 patients older or equal to 18-year-old (Fig. 1); 137 patients underwent MRI under GA more than once. Population characteristics are reported in Table 1. 18 procedures were cancelled for high anesthesiologic risk, 50 MRI were performed with no anesthesia,1389 MRI were performed under GA.

**Table 1:** Characteristics of the enrolled population

1457 Anesthesiologic records	
Age, median [IQR], years	4.0 [2.0–7.0]
Males, No. (%)	891 (61.2%)
Weight, median [IQR], kg	17.0 [12.0–24.9]
ASA score, No. (%)	
1	1267 (87.0)
2	171 (11.7)
3	19 (1.3)
4	0 (0)
5	0 (0)
Under chronic pharmacological treatment, No. (%)	475 (32.6)
Diagnostic category, No. (%)	
Static encephalopathies	492 (33.8)
Autistic spectrum disorders	164 (11.3)
Intellectual disabilities	93 (6.4)
Metabolic / evolutive encephalopathies	66 (4.5)
Epileptic encephalopathies	64 (4.4)
Neuromuscular diseases	63 (4.3)
Other psychiatric disorders	49 (3.4)
Migraine	27 (1.8)
Others	418 (28.7)
Missing data	21 (1.4)

*IQR*: Inter-quartile range

### Standardized sevoflurane-based NORA: applicability and features

Characteristics of the 1389 GA are reported in Table 2. 92.9% of anesthesia were performed as standardized. Sevoflurane was used as the induction drug in 93.3% of cases at a median dose of 8.0% (IQR 2.5 to 8.0). Much less frequently, propofol (4.7%), midazolam (1.3%), ketamine (0.6%) or thiopental (0.1%) were used. A standard premedication with midazolam IM with a median dose of 0.10 [0.09 to 0.11] mg*kg^− 1^ was added in 81.0% of cases (Table 2).

**Table 2:** Characteristics and complications of the MRI conducted under general anesthesia

1389 MRI conducted under general anesthesia	
Premedication, No. (%):	1134
Midazolam	1126 (99.3)
Atropine	4 (0.3)
Ketamine	2 (0.2)
Midazolam + atropine	1 (0.1)
Midazolam + atropine + ketamine	1 (0.1)
Induction, No. (%):	
Sevoflurane	1296 (93.3)
Propofol	66 (4.7)
Midazolam	18 (1.3)
Ketamine	8 (0.6)
Thiopental	1 (0.1)
Maintenance, No. (%):	
Sevoflurane	1314 (94.6)
Propofol	51 (3.7)
Midazolam	6 (0.4)
Ketamine	5 (0.4)
Thiopental	1 (0.1)
None	12 (0.8)
Spontaneous breathing, No. (%)	1262
Oral cannula	334 (26.5)
Cervical collar	809 (64.1)
Mechanical ventilation, No. (%):	127
LMA	126 (99.2)
Oro-tracheal tube	1 (0.8)
Anesthesia length, median [IQR], minutes	50 [40–60]
Recovery length, median [IQR], minutes	15 [10–20]

*MRI* Magnetic resonance imaging; *SpO*_*2*_ Pulsed-oxygen saturation; *NIBP* Non-invasive blood pressure; *EtCO*_*2*_ End-tidal carbon dioxide; *Et-sevoflurane* end-tidal sevoflurane; *EKG* electrocardiogram; *LMA* Laryngeal mask airway; *IQR* Inter-quartile range. *PONV* Perioperative nausea and vomiting

Standardized sevoflurane-based anesthesia was significantly less used in children affected by neuromuscular diseases (61.4 vs. 94.7%; *p* < 0.001) and required significantly less mechanical ventilation when compared to the other pharmacological approaches (8.6 vs. 16.2%; *p* = 0.012).

### Anesthesia features and management

Overall, in 96.7% of cases a mono-pharmacological approach (i.e. the same anesthesiologic drug for both induction and maintenance) was preferred (sevoflurane 96.0%, propofol 3.0%, ketamine 0.4%, midazolam 0.5%, thiopental 0.1%).

Among the 9.1% of ventilated patients, only one was intubated, all the others received an LMA. Patients of male sex (10.6 vs. 6.8%; *p* = 0.016), with ASA score > 1 (16.3 vs. 8.1%; *p* < 0.001), undergoing a non-sevoflurane-based anesthesia (16.2 vs. 8.6%; *p* = 0.012) and affected by neuromuscular disease (22.8 vs. 8.6%; p < 0.001) were significantly more frequently ventilated.

Overall, additional procedures (lumbar puncture, venous/arterial blood sample, other imaging, evoked acoustic potentials, muscular biopsy, electromyography) were associated in 16.9% of cases.

Median anesthesia length was 50 (IQR 40 to 60) minutes. Median time for full recovery was 15 (IQR 10 to 20) minutes; it was not affected by the length of GA or patients’ characteristics but was significantly longer in mechanically ventilated patients (20 (IQR 15 to 30) vs. 15 (IQR 10 to 20) minutes; *p* < 0.0001) and in patients with respiratory complications (20 (IQR 15 to 25) vs. 15 (IQR 10 to 20) minutes; *p* = 0.0236).

### General and respiratory complications

We registered 86 complications (6.2%): 43 respiratory complications (50.0%), 13 post-operative nausea and vomiting (15.1%), 10 hiccup (11.6%), 8 agitation (9.3%), 5 seizures (5.8%), 4 bradycardia (4.7%), 1 extravasation of glucose 5% (1.2%), 1 prolonged awakening (1.2%) and 1 inadequate sedation (1.2%). Among the 43 respiratory complications, we observed 23 airways obstructions (53.5%), 10 laryngospasms (23.3%), 5 depressions of the central respiratory drive (11.6%), 3 bronchospasms (7.0%) and 2 cough and hyper-salivation (4.7%).

We found statistically significant differences for general complications in ASA score, age, sex, the choice of a multi-pharmacological anesthesia and anesthesia length superior to 50 min, and for respiratory complications in ASA, a diagnosis of neuromuscular diseases and season autumn-winter (Table 3). A lower frequency of general complications in sevoflurane-based NORA is also observed, although it does not reach the statistical significance (5.9 vs. 10.1%; *p* = 0.095).

**Table 3:** Associations between general and respiratory complications and population characteristics

	General complications development			Respiratory complications development		
	Yes (86)	No (1303)	P^a^	Yes (43)	No (1346)	P^a^
ASA score > 1 (No. (%))	20 (23.3)	158 (12.1)	**0.003**	12 (27.9)	166 (12.3)	**0.003**
Age Median [IQR], months	54 [36–96]	48 [27–84]	0.050	60 [36–96]	48 [27–84]	0.071
Weight Median [IQR]), kg	18.6 [12.5–27.0]	16.6 [12.0–24.0]	0.170	18.9 [12.0–24.0]	17.0 [12.0–24.2]	0.356
Male sex No. (%)	62 (72.1)	784 (60.2)	**0.028**	32 (74.4)	814 (60.5)	0.065
Neuromuscolar diseases No. (%)	5 (5.8)	52 (4.0)	0.409	5 (11.6)	52 (3.9)	**0.028**
Premedication No. (%)	1063 (81.6)	71 (82.6)	0.821	34 (79.1)	1100 (81.7)	0.658
Multipharmacological anaesthesia No. (%)	7 (8.1)	39 (3.0)	**0.010**	0 (0.0)	46 (3.4)	0.218
Anaesthesia length > 50 min No. (%)	67 (77.9)	875 (67.2)	**0.039**	35 (81.4)	907 (67.4)	0.053
Season autumn-winter No. (%)	47 (54.7)	633 (48.6)	0.276	28 (65.1)	652 (48.4)	**0.031**

^a^*P* value for Pearson Chi-squared test and Wilcoxon rank-sum test

*ASA* American society of anesthesiologists; *IQR* Inter-quartile range

The level of statistical significance at 0.05 is set in bold

Table 4 shows the results of crude and adjusted ORs for general complications and respiratory complication development. The penalized logistic regression used as independent variables for general complications ASA score, multi-pharmacological anesthesia and anesthesia length superior to 50 min and for respiratory complications ASA score, presence of a neuromuscular disease, anesthesia length superior to 50 min and season autumn-winter; they were adjusted for age and sex. The risk for general complications increases with ASA score > 1 (OR 2.22, 95 CI% 1.30 to 3.77, *p* = 0.003), in male patients (OR 1.73, 95 CI% 1.07 to 2.81, *p* = 0.025) and in patients undergoing anesthesia with more than one drug (OR 2.98, 95 CI% 1.26 to 7.06, *p* = 0.013). For respiratory complications, the risk increases with ASA score > 1 (OR 2.34, 95 CI% 1.19 to 4.73, *p* = 0.017), in autumn-winter season (OR 2.01, 95 CI% 1.06 to 3.78, *p* = 0.030) and in patients affected by neuromuscular diseases (OR 3.18, 95 CI% 1.20 to 8.41, *p* = 0.020).

**Table 4:** Crude and adjusted OR and 95% CI general and respiratory complications

	General complication development						Respiratory complications development					
	OR	95% CI	p	Adj. OR	95% CI	p	OR	95% CI	p	Adj. OR	95% CI	p
ASA score > 1	2.19	1.29–3.73	**0.003**	2.22	1.30–3.77	**0.003**	2.75	1.38–5.48	**0.003**	2.34	1.19–4.73	**0.017**
Age (months)	1.01	1.00–1.01	0.061	1.03	0.97–1.08	0.281	1.01	1.0–1.01	0.086	1.06	0.98–1.13	0.099
Weight (Kg)	1.01	0.99–1.02	0.506				1.00	0.98–1.03	0.855			
Male sex	1.71	1.05–2.78	**0.028**	1.73	1.07–2.81	**0.025**	1.86	0.95–3.81	0.065	1.86	0.94–3.71	0.074
Neuromuscular diseases	1.49	0.58–3.82	0.409				3.27	1.24–8.68	**0.012**	3.18	1.20–8.41	**0.020**
Premedication	1.07	0.60–1.90	0.821				0.85	0.40–1.79	0.658			
Multi-pharmacological anesthesia	2.87	1.24–6.64	**0.010**	2.98	1.26–7.06	**0.013**	–	–	–	–	–	–
Non sevofluorane-based anesthesia	1.80	0.90–3.60	0.094				0.98	0.30–3.22	0.969			
Anesthesia length > 50 min	1.73	1.02–2.91	**0.039**	1.62	0.96–2.73	0.068	2.12	0.97–4.61	0.053	1.91	0.89–4.10	0.097
Season autumn-winter	1.28	0.82–1.98	0.276				1.99	1.05–3.76	**0.031**	2.01	1.06–3.78	**0.030**

*IQR* Inter-quartile range; *OR* Odds ratio; *Adj. OR* Adjusted odds ratio;

*CI* Confidence interval

The level of statistical significance at 0.05 is set in bold

In 35 cases, respiratory complications required an active intervention by the anesthesiologist; in 66.7% of cases this consisted in the placement of LMA. We observed no major complications compromising the patient’s outcome or requiring admission to ICU; 4 respiratory complications determined the definitive interruption and reschedule of MRI (0.29% of the 1389 GA).

## Discussion

The main findings of this retrospective cohort study are: 1. A standard approach with inhaled sevoflurane-based anesthesia could be applied in a high percentage of MRI sedation in pediatric patients affected by neuropsychiatric disorders. 2. With a standardized approach, anesthesia complications’ rate in this specific population is low and similar or lower to what reported in literature on general pediatric population. 3. Risk factors for general and respiratory complications could be identified.

Procedural anesthesia in children, performed outside the operating room, concerns an increasing number of patients every year, since required to reduce pain, stress and discomfort associated to health care procedures. Initially performed without specific guidelines, first recommendations appeared in 1985 [2] to standardize the technique and establish minimal safety requirements, after the death of 3 patients following deep sedation in dentistry. In the following years, many guidelines have been emanated by different international societies, until the publication of shared recommendations, recently updated in 2016 [3–5]. Guidelines deeply detail the most appropriate level of sedation, monitoring, data record and dedicated personnel. No clear indications on the most appropriate drugs are published and each hospital developed its own protocol for pediatric sedation, thus leading to a variety of clinical practices.

In the context of MRI, the goal of anesthesia is to facilitate this painless procedure providing immobility, safety and comfort; it has been shown that adequate sedation and airways management significantly improves image quality [1], thus reducing the need of repeated exams. Specific issues of MRI sedation are lack of visibility, unavoidable distance between patient and anesthesiologist, lack of access to the patient, need of MRI-compatible tools. Since even the slightest movement can cause artefacts, deep sedation or general anesthesia is required.

Previous studies focused on specific pharmacological approaches for NORA in children. Chloral hydrate has been widely used in patients affected by neuropsychiatric disorders undergoing MRI [6–9]; however, it is poorly predictable and requires in the large majority of cases to be integrated with one or two additional sedative drugs. This implicates a high sedation failure and exam interruption rates (6–20% and 2.3–6% respectively) [6–9]; anesthesia respiratory complications’ rate varies from 4 to 9% [6–9]. Moreover, a recovery time of around 30 min is reported [8]. The association of pentobarbital and chloral hydrate has also been proposed but is also affected by high sedation failure (6%) and complications’ rate (9%). Dexmedetomidine has been more recently introduced to pediatric sedation [10, 11], but it is affected by extremely high sedation failure rate when used alone (above 90%). Its low rate of respiratory complications (0.3%) is counterbalanced by a significantly high risk of cardiovascular side effects (hypotension 9.5%, bradycardia 20.3%) [10]. Finally, a recovery time of around 40 min is reported [10]. Benzodiazepines are also affected by high frequency of inadequate sedation and sedation failure [7]. Propofol presents many advantages, such as short duration, predictable response, prompt awakening; it’s the most frequently used drugs in many American and Canadian centers [12]. However, previous studies in general pediatric populations report a relatively high frequency of respiratory complications (10.6–31.9%) [13, 14]. A low dose propofol anesthesia combined with nalbuphine has been proposed in a mixed pediatric population with a very low rate of respiratory complications (1%) [15]. A recovery time of around 30 min is described [14]. Finally, it requires a previously placed venous catheter and an MRI compatible infusion system.

Halogenates have been successfully used in newborns and infants [16, 17]; they provide multiple advantages, such as rapid induction, optimal maintenance, speedy recovery and minimal incidence of complications. De Sanctis et al. [17] safely used a mix of sevoflurane 1.5–2%, oxygen and nitrous oxide in newborns and infants younger than 1-year-old with a rate of general and respiratory complications of 2 and 1.9%, respectively.

We therefore systematically applied in the last ten years a standardized approach with sevoflurane-based anesthesia in children with neuropsychiatric disorders undergoing MRI. This standard approach could be used in 92.3% of cases and was associated to a significantly lower rate of mechanical ventilation when compared to all the other pharmacological approaches combined. Interestingly, a mono-pharmacological NORA was also a protective factor for overall complications’ development and in 96.0% it consisted in a sevoflurane-based NORA. Despite the remarkable length of anesthesia, median recovery time was very short when compared to literature, possibly thanks to the speedy awakening provided by sevoflurane and the possibility to avoid mechanical ventilation in more than 90% of patients.

Overall, spontaneous breathing was maintained along the entire MRI exam in a high percentage; airways patency assessment was mainly based on thoracic excursion and EtCO_2_ waves analysis, thus confirming the need for a complete respiratory monitoring. However, advanced airways management was required in 9.1% of cases, confirming the need for adequately trained personnel.

Finally, children affected by neuropsychiatric disorders may present specific anesthesiologic issues [24–30]. Previous studies suggest that children with developmental disabilities have a threefold increased incidence of desaturation compared to others [18], possibly due to a smaller upper airways’ diameter [20] or to the presence of neuromuscular diseases involving respiratory muscles [24–30]. In our population with a standardized anesthesiologic approach, we observed a low rate of complications, and no severe complications compromising the patient’s outcome or requiring admission to ICU. General and respiratory complications’ rates were respectively 6.2 and 3.1%, in range or even lower when compared to similar studies in literature in mixed children populations. ASA score was confirmed to be a reliable parameter in identifying high-risk patients. Interestingly, while younger children are in general considered at higher anesthesiologic risk, we recorded a higher median age in patients developing general complications. However, age didn’t result to be an independent risk factor for general or respiratory complications. Concerning anesthesia management, premedication didn’t seem to impact on complications’ development; a mono-pharmacological approach for induction and maintenance seems on the contrary to be a protective factor and was in our case mainly a sevoflurane-based NORA. Longer anesthesia, which may correspond to longer MRI exam or to the presence of mechanical ventilation or associated procedures, increased of 1.62 the risk of complications’ development. Finally, patients with neuromuscular diseases were confirmed to have a higher risk of respiratory complications. The identification of risk factors in this population may allow anesthesiologists trying not to overlap them, when possible.

### Limitations

The main weakness of this study is the retrospective design: anesthesiologic records were compiled manually and parameters were reported every 10 min. However, we had no missing data on pharmacological anesthesia management. Moreover, the study was not built to compare different pharmacological approaches. Future studies should focus on the comparison of different pharmacological approaches in specific neuropsychiatric classes.

Finally, IM midazolam administration could be easily replaced by less invasive approaches such as nasal administration.

## Conclusion

A standardized sevoflurane-based NORA for pediatric patients with neuropsychiatric disorders undergoing MRI is highly feasible and safe. In this population, children with ASA score > 1, of male gender, undergoing prolonged procedures in winter-autumn months and with neuromuscular diseases should be considered at higher risk of general and respiratory complications.

## Data Availability

The datasets used and/or analysed during the current study are available from the corresponding author on reasonable request.

## Abbreviations

ASA: American Society Anesthesiology
CI: Confidence Interval
GA: General anesthesia
IM: Intramuscular
IQR: Inter-quartile range
LMA: Laryngeal mask airways
MRI: Magnetic Resonance Imaging
NORA: Non operating room anesthesia
SD: Standard deviation
